# Entomopathogenic Fungi on *Hemiberlesia pitysophila*

**DOI:** 10.1371/journal.pone.0023649

**Published:** 2011-08-25

**Authors:** Chengqun Lv, Baoling Huang, Mengji Qiao, Jiguang Wei, Bo Ding

**Affiliations:** Forestry College, Guangxi University, Nanning, China; Ghent University, Belgium

## Abstract

*Hemiberlesia pitysophila* Takagi is an extremely harmful exotic insect in forest to Pinus species, including *Pinus massoniana*. Using both morphological taxonomy and molecular phylogenetics, we identified 15 strains of entomogenous fungi, which belong to 9 genera with high diversities. Surprisingly, we found that five strains that were classified as species of *Pestalotiopsis*, which has been considered plant pathogens and endophytes, were the dominant entomopathogenic fungus of *H. pitysophila*. Molecular phylogenetic tree established by analyzing sequences of ribosomal DNA internal transcribed spacer showed that entomopathogenic *Pestalotiopsis* spp. were similar to plant *Pestalotiopsis*, but not to other pathogens and endophytes of its host plant *P. massoniana*. We were the first to isolate entomopathogenic *Pestalotiopsis* spp. from *H. pitysophila*. Our findings suggest a potential and promising method of *H. pitysophila* bio-control.

## Introduction

Insect pathogenic fungi or entomopathogenic fungi (EPF) are the fungi capable of invading, parasitizing, and causing insects sick or death. More than 60% of naturally-occurred insect epidemics are caused by pathogenic fungi, including EPF, one of the major controlling nature factor of insect populations [1]. About 750 to 800 EPF species in more than 100 genera have been recorded worldwide [2], and new taxa are constantly being discovered. However, only *Lagenidium giganteum*, *Beauveris bassiana*, *B. brongniartii*, *Metarhizium anisopliae*, *Paecilomyces fumosoroseus* and *Verticillium lecanii* have been commercially used as pesticides so far [3].

With increased human awareness of environmental protection, studies using combined methods of community ecology and insect mycology on EPF biodiversities including species diversities, community structures, and ecological distributions in different habitats, especially those forest ecosystems with large number of EPF species and qualities, have attracted more and more attention of mycologists. Tzean et al. [4] found 24 genera and 66 species of EPF in Taiwan. Harry et al. [5] investigated EPF of plant beetles in Worcestershire, England. Miroslav et al. [6] reported biodiversity of *Geosmithia* spp. of bark beetles in the Mediterranean region. Chen et al. [7] and Li et al. [8] investigated eco-diversities of EPF in Langya Mountain, Anhui Province, and in Shanxi Province, China, respectively. Maurer et al. [9] analyzed the genetic diversity of *B. bassiana* and its relation to its host insects. These studies laid scientific foundation not only for understanding EPF's biological characteristics, relationship to hosts and natural distributions, but also for reasonable conservation and sustainable utilization of these natural resources.

Entomogenous fungi in *Pinus massoniana* forest have been reported. For example, Wang et al. [10] studied their community structure in Beauveria-inoculated *P. massoniana* forest. They found 7 entomogenous fungal species in 4 genera and explored the population dynamics of *B. bassiana*. Zhang et al. [11] studied host diversity and dynamics. However, so far there has been no report on either entomogenous or entomopathogenic fungi of *Hemiberlesia pitysophila* Takagi.

*H. pitysophila* is an exotic, extremely harmful quarantine pest for pines including *P. massoniana* [12]. Since it was imported in Guangdong Province, China, at early 80s of the 20th century, *H. pitysophila* has been widely spread in Southern China and seriously damaged China's *P. massoniana* forest. An outbreak may cause a decline of pine volume growth by 2.548 m^3^, which is about 3.2-fold of the damage caused by pine caterpillars. The forest may die out in 3 to 5 years [13]. During the past 30 years, natural parasitic enemies as well as other chemical and physical methods have been used in controlling *H. pitysophila*. However, their effectiveness is not satisfactory. In this study, we, for the first time, noticed a peculiar natural death of *H. pitysophila* in the epidemic areas of Guangxi province and then isolated and cultured entomogenous fungi from naturally died *H. pitysophila*, performed morphological taxonomic and molecular systematic identification, and found five entomopathogenic species of *Pestalotiopsis*, which were the most frequent and virulent fungi to *H. pitysophila*.

## Materials and Methods

### Cadaver collection of *H. pitysophila*

Cadavers of *H. pitysophila* were collected from coniferous leaves of *P. massoniana* in epidemic areas. The cadavers were sealed in kraft paper bags on the spot and immediately disinfected to isolate fungi in the laboratory. Three batches were collected from Cenxi, Beiliu and Luchuan county, and 300∼400 cadavers of *H. pitysophila* were randomly sampled from each batch.

### Isolation of entomogenous fungi and their taxonomic identification by morphology

The cadaver were surface sterilized with 75% ethanol for 15 seconds and repeatedly washed with sterile water. After the remaining water was absorbed with sterile filter paper, each cadaver from different batch were separately placed in 9-cm culture dishes containing sterilized potato dextrose medium (PDM) and cultured at 28°C till fungal colonies appeared. Hyphae from single colony were transferred to a new PDM plate and cultured at 28°C. After each strain was purified by inoculation of amerospore, they were preserved on slant at 4°C.

The morphology of fungi cultured on PDM plates was observed and used for taxonomic identification according to Wei [14], Barnett and Hunter [15].

### Identification of entomogenous fungi by Molecular taxonomy

Five days after cultured on PDM, DNA of all fungal strains was isolated as previously reported [16] and used as template to amplify internal transcribed spacer (ITS) of rDNA as reported previously [17] using primers ITS4 (5′-TCCTCCGCTTATTGATATGC-3′) and ITS5 (5′-GGAAGTAAAAGTCGTAACAAGG-3′). All ITS amplicons were sequenced by Sangon Biotech (Shanghai) Co., Ltd. The sequences were blasted against the Genbank nucleotide sequence database (http://blast.ncbi.nlm.nih.gov/) and the most similar sequences were selected to compare with reference sequences from the Genbank using ClustalX (1.81) software. Fungal species were determined by the highest similarity of ITS sequences to known strains. Phylogenetic tree of *Pestalotiopsis* spp. was established using software MEGA 4. *Diaporthe phaseolorum*, *Pleospora herbarum* and *Hypocrea schweinitzii* were used as outgroup control.

### Virulence of *Pestalotiopsis* spp

The toxicity of five isolated *Pestalotiopsis* spp. strains that appeared most frequently in *H. pitysophila* was examined using randomized block experiments with three repeats. Water was used as control and blocks were separated with protected zones. In brief, the strains were individually inoculated on liquid PDM and cultured at 28°C on a shaker for 72 h. The five culture suspensions were diluted and thoroughly mixed with 5-fold sterile water individually, Number of blastospore was counted under a microscope and the final concentration of each mixture was 10^8^ cells/L, which is the normal concentration of *Beauveris bassiana* blastospore for treating pine caterpillars. Five mixtures were then individually sprayed on the crown of 5 trees of *P. massoniana* infected with *H. pitysophila*.

### Sample collection and Mortality calculation of *H. pitysophila*

**Figure 1:**
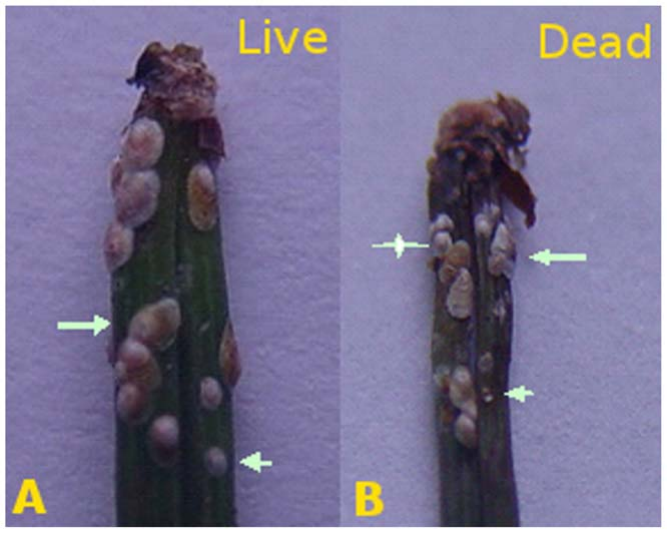
The live and dead *H. pitysophila* were identified under a magnifier. Live insects were shown in A and dead ones in B. Both adults (arrow) and nymphs (arrow head) were observed in one leaf. Fungi were found on most dead ones (asterisk).

Seven days after the spray, the dead and live adult *H. pitysophila* in 30 needle fascicles from different locations of each tree were counted to calculate the mortality of *H. pitysophila* for each strain. The live and dead *H. pitysophila* were identified under a magnifier. Bodies of live insects were shiny and plump (Figure 1A), whereas the bodies of dead ones were dark and shrivelled, and they easily fell off the leaves (Figure 1B). At least 100 live or dead bodies in 30 needle fascicles were counted. The mortality was calculated as follows:

Mortality = Dead/Total body counts.
We also adjusted the mortality to:
Adjusted mortality = Calculated mortality – Natural mortality.

The natural mortality was defined as the mortality recorded without any *Pestalotiopsis* spp. treatment, while calculated mortality was defined as the mortality recorded with *Pestalotiopsis* spp. treatment. The difference of the adjusted mortality among the five strains was tested using one-way ANOVA. A p<0.05 was considered statistically significant.

## Results

### Morphological taxonomy of entomogenous fungi on *H. pitysophila*

Fungi isolated from the cadavers of naturally died *H. pitysophila* were preliminarily classified into No. 1 to 15 strains based on the morphological characteristics of their colonies and further identified by microscopic observations, as shown in Table 1.

**Table 1:** Taxonomic identification of entomogenous fungi of *Hemiberlesia pitysophila* based on their morphology.

*Strain No.*	*Morphological characteristics*	*Preliminary identification*
1	This strain had fewer hyaline or light hyphae that frequently become bright dark-brown with age and undergo transformation to form chains of numerous, single-celled, ovoid-shaped conidia. Some conidia were produced through budding.	Genus *Aureobasidium*
2	Same as No. 1	
3	This strain had dark, disc- or cushion-shaped acervuli containing black, slimy spore masse and 5-celled, oval to spindle-shaped conidia. Their apical and basal cells were hyaline, while the median three cells were olivaceous; the upper two were the same as or slightly darker than the lower one. The apical cell was cylinder- or cone-shaped and slightly narrower than the basal cell. The fungi had flagella on its top and 0–1 root on its base.	Genus *Pestalotiopsis*
4	This strain had hyaline, single-celled, oval conidia with many lateral or paired, but not verticillati branches and phialides on the tips of branches. It was distinct due to its rapid growth and compact or loose tufts in shapes of green.	Genus *Trichoderma*
5	This train had dark, mostly simple, very short or elongated conidiophores typically with simple or branching conidium chains. Conidia were dark in conical, elliptical, oval shapes and typically separated by both diaphragm and mediastinum. Individual conidiophore arises directly from substrata forming bushy heads consisting conidial chains, occasionally single, simple and branching appendages.	Genus *Alternaria*
6	This train had nearly spherical conidiophore mother cells that produced mostly simple, hyaline conidiophores with elongated base except thick, dark, septate conidiophores. Its conidia were dark, 1-celled with spindle, oval shape and curved tip, and attached to lateral or and top of the conidiophores, often with a small bud scar at one side.	Genus *Arthrinium*
7	This strain had pycnidia in globose, ovoid, or flask-shaped, leathery or charry, black or dark brown color. It split in small holes, bears conidia on conidiophores or embeded in the substrata of pycnidia.	Family *Sphaeropsidaceae*
8	The strain had 1-celled, elliptical or ovoid, hyaline conidia; waxy, disc-butterfly or pad shaped acervuli close to the tips of simple and elongated conidiophores, typically with dark spines or bristles in the middle or around the rim.	*Genus Colletotrichum*
9	The strain was in the incompletely phase of *Diaportheres*, with simple conidiophore and 1-celled, ovoid or spindle-shaped, hyaline conidia. Its dporodochidia were globose, prominent, dark, split in small holes and embeded in substrata of pycnidia.	Genus *Diaportheres*
10	Same as No. 3	
11	Same as No. 3	
12	Same as No. 3	
13	Same as No. 3	
14	Same as No. 3	
15	This strain had unbranched, solitary, erect conidiophores formed directly on the hyphal tips, the hyphal ropes or both. At the apices of the conidiophores were the hyaline conidia of 3–6×1.5–3 µm in size.	Genus *Acremonium*

### Taxonomic identification of entomogenous fungi on *H. pitysophila* by ITS sequencing

Renske et al. [18] have identified species in soil ectomycorrhizal fungal community using molecular rDNA ITS sequencing method. After blasting in the Genbank, they proposed that fungi with ITS sequences similarity ≥99% could be considered as the same species; with sequence similarity from 95% to 99% could be identified as same genus; with sequence similarity ≤95% could be identified as family. In this study, we obtained rDNA ITS sequences from the 15 fungal strains. BLAST analysis (Table 2) of these ITS sequences showed that all the fungi had ITS sequences with 98% to 100% similarity to known fungi in the GenBank. Among them, nine strains were identified to six species of four genera; five strains were identified to genus; and one strain was only identified to family, similar to uncultured Ascomycete, but not to the species of Sphaeropsidaceae.

**Table 2:** Taxonomic identification of entomogenous fungi of *Hemiberlesia pitysophila* based on ITS sequences.

*Isolated strains*	*Length of ITS region (bp)*	*Closest species*			
		Species/Strain/Sequence	GenBankAccession No	Value	Similarity
1	576	*Aureobasidium pullulans* strain B8	FJ216455.1	1029	99%
2	607	*Aureobasidium pullulans*	AY225166.1	1083	100%
3	634	*Pestalotiopsis vismiae*	EU273510.1	1260	100%
4	632	*Trichoderma atroviride* strain DAOM	EU280133.1	1168	100%
5	600	*Alternaria* spp.	EF432260.1	1105	100%
6	608	*Arthrinium* spp.	AB505426.1	1096	99%
7	563	Uncultured ascomycete	EU489900.1	1002	98%
8	586	*Colletotrichum gloeosporioides*	AY266393.1	1083	100%
9	566	*Diaporthe eres*	FJ478132.1	1026	99%
10	631	*Pestalotiopsis disseminata*	AB251918.1	1116	99%
11	622	*Pestalotiopsis vismiae*	EU273510.1	1249	100%
12	631	*Pestalotiopsis vismiae*	FJ481027.1	1105	98%
13	548	*Pestalotiopsis vismiae*	EU273510.1	1078	100%
14	602	*Colletotrichum* spp.	DQ780418.1	1083	99%
15	578	*Acremonium* spp.	FJ770373.1	1018	99%

The taxonomic identification of entomogenous fungi on *H. pitysophila* except for the No. 7 strain by ITS sequencing (Table 2) was in agreement with the classification based on morphological characteristics (Table 1).

### Composition of entomogenous fungi

Taxonomic identification of entomogenous fungi on *H. pitysophila* by ITS sequencing and morphological characteristics indicated that the 15 isolated entomogenous fungi belong to 2 classes, 4 orders, 5 families and 9 genera. As shown in Table 3, entomogenous fungi on *H. pitysophila* were diverse in both number and type, and 93.3% of them belonged to Class Fungi imperfecti; only 16.7% belonged to Class Ascomycetes. Orders of Moniliaceae and Melanconiaceae accounted for 46.7% and 40%, respectively. Families of Moniliaceae and Melanconiaceae accounted for 46.7% and 26.7%, respectively. Species of *Pestalotiopsis* were the dominant ones, accounting for 33.3% of the total strains.

**Table 3:** Group composition of entomogenous fungi of *Herminerlesia pitysophila.*

*Class*	*Order*	*Family*	*Genus*	*No. of strain*
Fungi imperfecti	Moniliales	Moniliaceae	*Aureobasidium*	2
			*Trichoderma*	1
			*Acremonium*	1
		Dematiaceae	*Alternaria*	1
			*Arthrinium*	1
	Melanconiales	Melanconiaceae	*Pestalotiopsis*	5
			*Colletotrichum*	2
	Sphaeropsidales	Sphaeropsidaceae	Unknown	1
Ascomycetes	Sphaeriales	Diaporthaceae	*Diaporthe*	1

### Phylogenetic tree construction of entomopathogenic *Pestalotiopsis*

**Figure 2:**
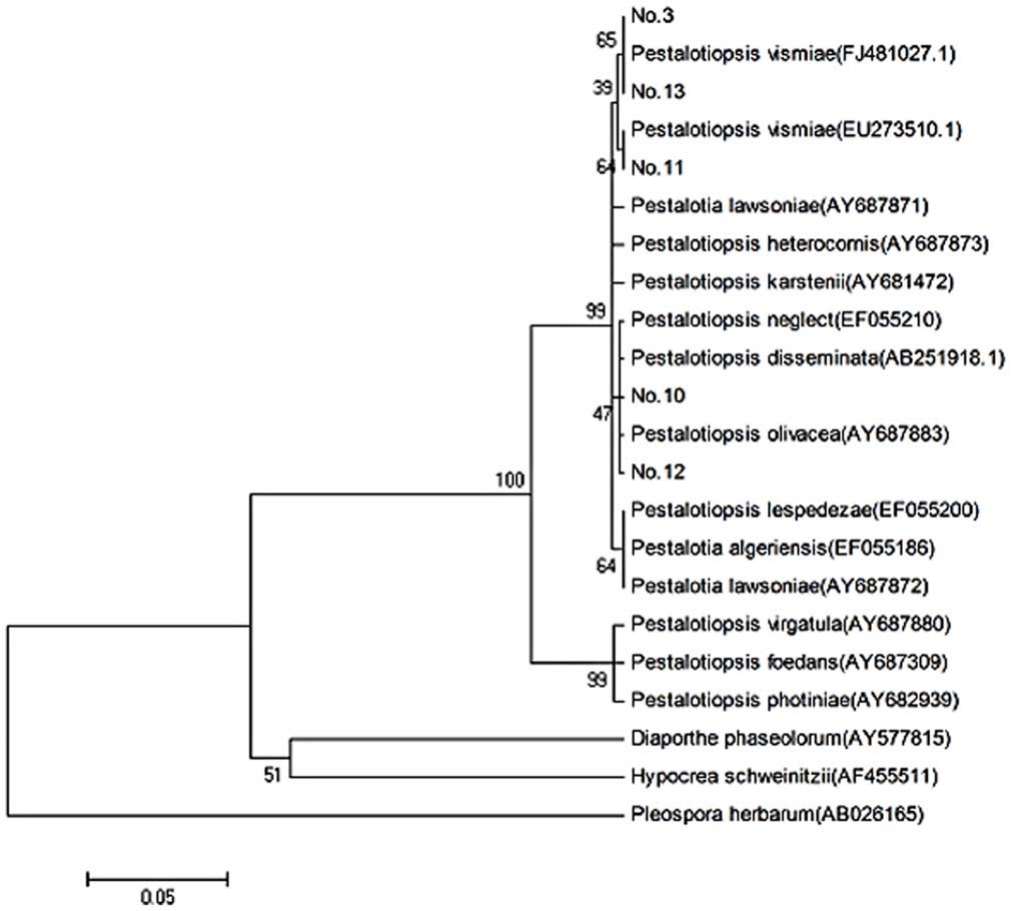
Established phylogenetic tree of Pestalotiopsis and its related genera based on ITS sequences. ITS sequences were aligned using ClustalX (1.81). The phylogenetic tree construction were conducted with neighbour-joining method packaged in software MEGA 4.0. Bootstrap = 1000. *Diaporthe phaseolorum*, *Pleospora herbarum* and *Hypocrea schweinitzii* were used as the outgroup. The Scale bar shows 5% nucleotide substitutions.

We found that No. 3, 10, 11, 12, and 13 fungi belonged to genus *Pestalotiopsis*, which was previously only isolated from plants themselves and considered as plant pathogens or endophytes [19]. The known AY682939 and AY687871 have been identified as pathogenic fungi for *P. massoniana*, while AY687880, AY687309 and AY681472 are endophytic fungi for *P. massoniana* [19]. To understand the relationship of entomogenous and plant pestalotiopsis, we established phylogenetic tree by comparing sequences of 5 strains with that of known *Pestalotiopsis* spp. in the GenBank (Figure 2). We found that strains No. 3, 11 and 13 were clustered with *P. vismiae* (FJ481027.1 and EU273510.1) on the same branch, and strains No. 10 and 12 were close to *P. olivacea* (AY687883) and *P. disseminata* (AB251918.1), indicating that no significant difference between entomopathogenic and plant *Pestalotiopsis* spp. in systematic development. The results also showed that the relationship of entomopathogenic *Pestalotiopsis* spp. was different from other pathogenic and endophytic species of *P. massoniana* except pathogen AY687871. Together, the results not only indicated that *Pestalotiopsis* spp. had insect host besides plant, but also excluded entomopathogenic *Pestalotiopsis* spp. were from infectious pathogens and endophytes of *P. massoniana*.

### Virulence of entomopathogenic fungi

Seven days after the five suspensions were individually sprayed uniformly on the crown of *P. massoniana* with *H. pitysophila*, the mortality of *H. pitysophila* was calculated. As shown in Table 4, the rectified mortality of *H. pitysophila* was between 67.61%∼74.89% and No. 10 strain treated group showed the highest mortality (74.89%), followed by 73.91% of No. 3 strain, 70.50% of No. 11 strain, 70.47% of No. 13 strain and, the lowest 67.61% of No. 12 strain. Compared with the natural mortality of 22.40%, the five tested strains of *Pestalotiopsis* showed remarkable protection of *P. massoniana* by killing *H. pitysophila*.

**Table 4:** Mortality of *Hemiberlesia pitysophila* in virulence test.

Strains	Repeat I		Repeat II		Repeat III		Average mortality	Average adjusted mortality
	Mortality	Adjusted mortality	Mortality	Adjusted mortality	Mortality	Adjusted mortality		
3	93.28	70.88	100.00	77.60	95.65	73.25	95.65	73.91
10	97.97	75.57	100.00	77.60	93.90	71.50	97.29	74.89
11	100.00	77.60	95.65	73.25	83.05	60.65	92.90	70.50
12	98.64	76.24	96.39	73.99	75.00	52.60	90.01	67.61
13	97.06	74.66	100.00	77.60	81.54	59.14	92.89	70.47
Control	22.4		22.4		22.4		22.4	

As shown in Table 5, one-way ANOVA analysis of the rectified mortality indicated that the tested 5 strains were not significantly different in lethal toxicity (F = 0.8397<F_0.05_ = 3.84), suggesting that all these five strains could be used to control *H. pitysophila* with the same efficacy.

**Table 5:** ANOVA of the adjusted mortality for five strains.

Source of differences	SS	df	MS	F	F crit
Intragroup	103.4911	4	25.8728	0.8397	F_0.05_ = 3.84
Intergroup	246.4814	8	30.8102		
Sum	838.2601	12			

## Discussion

*H. pitysophila* were imported to China at early 80s of the 20th century from its initial habitation Japan and Taiwan. Afterwards, they widely spread in Southern China causing disasters in large areas. So far, its entomopathogenic fungi have not been systematically studied. In this study, 15 entomogenous fungi were isolated from *H. pitysophila* and their taxonomies were analyzed by morphological and ITS sequence-based molecular methods. We found 5 strains of fungi were entomopathogenic, indicating that there are antagonistic microbial species in the new habitation of *H. pitysophila* and it is possible to control the spread of *H. pitysophila* infestation.

Currently, the most commonly studied and used pesticidal entomogenous fungi in China and worldwide are *L. giganteum*, *B. bassiana*, *B. brongniartii*, *M. anisopliae*, *P. fumosoroseus* and *V. lecanii* [3] This study found nine entomogenous fungal genera (species) including *Pestalotiopsis*, Aureobasidium, Trichoderma, Acremonium, Alternaria, Sphaeropsidaceae, Colletotrichum and *Diaporthe eres*. Species of Acremonium have been previously found in Dabie and Langyashan Mountains of Anhui Province, China [7] [20] and species of Alternaria have been found in Dabie and Langyashan Mountains of Anhui Province, Lishan National Nature Reserve and Pangquangou National Nature Reserve [7], [20]–[22]. The main hosts of Acremonium in Langyashan area are Coleoptera, *Fulgoroidea* spp. and *Cryptorymp* spp. [7], and the host of Alternaria genus in Pangquangou National Nature Reserve [22] is Coleoptera. To our knowledge, we were the first to isolate entomopathogenic *Pestalotiopsis* spp. from insects.

*Pestalotiopsis* has been considered as plant pathogens for more than a century [19], [23], [24]. Since Espinosa-Garcia et al. [25] first reported *Pestalotiopsis funerea* was the major endophytic fungus of *Sequoia sempervirens*, 46 species of Pestalotiopsis have been reported as endophytes of 25 plants [26]–[29]. Pestalotiopsis, Alternaria, *Acremonium pullulans* and Trichoderma are important component of endophytic fungi of *P. massoniana* [30]. This is the first report of *Pestalotiopsis* spp. as dominant entomopathogenic fungi of *H. pitysophila*.

Morphological comparison, inoculation test and molecular systematical analysis have indicated that *Pestalotiopsis* has no apparent host specificity [31]. Pathogenic *Pestalotiopsis* and endogenous *Pestalotiopsis* have no fundamental difference. Same *Pestalotiopsis* could be pathogenic to one plant and endophytic to another plant [31]–[34]. Numerous studies have shown that the relationship between plants and *Pestalotiopsis* spp. can be parasitic, symbiotic and saprophytic [35]. However, the virulence tests showed that as dominant entomopathogenic fungi, *Pestalotiopsis* spp. is very toxic to *H. pitysophila*, but not to its host *P. massoniana*, indicating *Pestalotiopsis* is somewhat specific for *H. pitysophila.* Our ITS-based phylogenetic analysis showed that *Pestalotiopsis* is not phylogenetically different from plant *Pestalotiopsis*, but different from pathogenic and endophytic *Pestalotiopsis* of *P. massoniana*, the host of *H. pitysophila*. Therefore, *Pestalotiopsis* can be used as antagonistic microbe to control *H. pitysophila*.

The biocontrol of *H. pitysophila* is important for the pine forest protection. Pan et al. [36] tried *Cladosporium cladosporioides* (Fresen.) G.A. de Vries, a common fungus found on *Kermes nawae* kuwana, to control *H. pitysophila*, yielding only a 20% mortality. Li et al. [37] used *Aspergillus parasilicus* Spere to control *H. pitysophila*, and got an unstable mortality ranging from 13% to 64%. In this study, we found *Pestalotiopsis* spp. could achieve 67% to 74% mortality, which makes the fungi potential candidates for controlling *H. pitysophila*. It is not clear why *Pestalotiopsis* spp. are specific for killing *H. pitysophila*. The future study may be focused on the micro-ecological environment and some specific metabolic pathways in *H. pitysophila*.

## References

[1] Charnley AK (1997). Entomopathogenic fungi and their role in pest control. Wicklow/soderstrom. The Mycota IV.. Environmental and Microbial Relationships.

[2] Kirk PM, Cannon PF, David JC, Stalpers JA (2001). Dictionary of the Fungi..

[3] Shah PA, Pell JK (2003). Entomopathogenic fungi as biological control agents.. Appl Microbiol Biotechnol.

[4] Tzean SS, Hsieh LS, Wu WJ (1997). Atlas of Entomopathogenic Fungi from Taiwan..

[5] Harry C, Whitehead PF, Whitehead (2005). Entomogenous fungi of arboreal Coleoptera from Worcestershire, England, including the new species. Harposporium bredonense.. Mycological Progress.

[6] Miroslav K, Martin K, Sylvie P (2007). Host range and diversity of the genus Geosmithia (Ascomycota: Hypocreales) living in association with bark beetles in the Mediterranean area.. Mycological Research.

[7] Chen MJ, Huang B, Wang M, Wu SZ, Fan MZ, Zou YD, Li ZZ (2007). Species diversity and seasonal change of entomogenous fungi in Langya Mountains Nature Reserve.. Chinese Journal of Applied Ecology.

[8] Li WY, He YC, Wang JM, Zhang ZG, Zhang XH (2003). Ecological diversity of entom ogenous fungi of three nature reserves in Shanxi Province.. Biodiversity Science.

[9] Maurer P, Couteaudier Y, Girard PA, Bridge PD, Riba G (1997). Genetic diversity of Beauveria bassiana and relatedness to host insect range.. Mycological Research.

[10] Wang B, Ding D, Fan M, Li Z (2005). Community structure of entomogenous fungi in Beauveria-inoculated Masson's pine forest.. Chinese Journal of Applied Ecology.

[11] Zhang YB, Wang XL, Fan MZ, Wang L, Li ZZ (2007). Diversity of entomogenous fungi and of the fungi in a Masson's their hosts and population dynamics pine plantation ecosystem.. Journal of Anhui Agricultural University.

[12] Huang ZY (2001). Pest risk analysis of H emibedesia pitysophila Takagi.. Entomological Journal of East China.

[13] Wu J (2004). Invasion actuality and Countermeasures against Alien Forest Pests in China.. Science & Technology Review.

[14] Wei JC (1979). Identification Handbook of Fungus..

[15] Barnett HL, Hunter BB (1977). Illustrated Genera of Imperfect Fungi (in Chinese)..

[16] Doyle JJ, Doyle J (1987). A rapid DNA isolation procedure for small quantities of fresh leaf tissues.. Phytochemistry Bulletin.

[17] Nnis MA, Gelfand DH, Sninsky JJ, White TJ (1990). PCR Protocols: A guide to methods and applications.

[18] Renske L, Paula L, Thom WK, Ellis H, Anna R, Karel W, Eric S (2003). Molecular Identification of Ectomycorrhizal Mycelium in Soil Horizons.. Applied and Environmental Microbiology.

[19] Wei JG, Tong X, Huang WH, Guo LD, Pan XH (2008). Molecular phylogenetic investigation on relationship of endophytic and pathogenic Pestalotiopsis species.. Journal of Zhejiang University (Agric1&Life Sci).

[20] Wang S, Liu J, Wang C, Fan M, Li Z (2004). Community diversity of entomogenous fungi in Dabie Mountains in Anhui.. Chinese Journal of Applied Ecology.

[21] Song DH, Song SM, Zhang ZG (2001). Study on the Resources and Pathogenicity of Entomogenous Fungi in Lishan National Nature Reserve.. Forest research.

[22] Li WY, He YC, Wang JM (2003). Study on the Resources of Entomogenous Fungi in Pangquangou National Nature Reserve.. Forest research.

[23] Lisa MK, Maile EV, Francis TZ (2006). Identification and characterization of Pestalotiopsis spp. Causing Scab Disease of Guava, Psidium guajava, in Hawaii.. Plant Disease.

[24] Jillian ML, Julie MS (1990). Diseases of Desmodium Species Review.. Tropical Grasslands.

[25] Espinosa-Garcia FJ, Langnheim JH (1990). The endophytic fungal community in leaves of a coastal redwood population-diversity and spatial patterns.. New Phytologist.

[26] Tejesvi MV, Tamhankar SA, Kini KR, Rao VS, Prakash HS (2009). Phylogenetic analysis of endophytic Pestalotiopsis species from ethnopharmaceutically important medicinal trees.. Fungal Diversity.

[27] Kumar DSS, Hyde KD (2004). Biodiversity and tissue-recurrence of endophytic fungi in Tripterygium wilfordii.. Fungal Diversity.

[28] Wei JG, Xu T, Guo LD, Liu AR, Zhang Y, Pan XH (2007). Endophytic Pestalotiopsis species associated with plants of Podocarpaceae, Theaceae and Taxaceae in southern China.. Fungal Diversity.

[29] Pedro WC, Gerard JMV, Groenewald JZ (2006). Eucalyptus microfungi known from culture. 1. Cladoriella and Fulvoflamma genera nova, with notes on some other poorly known taxa.. Studies in Mycology.

[30] Zeng FY (2006). Population dynamics of Bursaphelenchus xylophilus with relationship to the inner habiting fungi of Pinus spp..

[31] Wei JG, Xu T, Guo LD, Pan XH (2005). Endophytic Pestalotiopsis species from Southern China.. Mycosystema.

[32] Wang Y, Guo LD (2004). Endophytic fungi II. New records from Pine in China.. Mycosystema.

[33] Wei JG, Xu T (2003). Pestalotiopsis karstenii, a new record of endophytic fungi from Camellia sasanqua in China.. Mycosystema.

[34] Guo LD (2002). Pestalotiopsis besseyi, A new record of endophytic fungi from Pine in China.. Mycosystema.

[35] Wei JG, Xu T, Pan XH (2006). Progress of research on taxonomy of Pestalotiopsis.. Journal of Guangxi Agric and Biol Science.

[36] Pan WY, Chen SL, Lian JH, Qiu HZ, Lan G (1989). A report of using *Cladosporium cladospo- rioides* to control *Hemiberlesia pitysophila*.. Forest Pest and Disease.

[37] Li LZ, Li YC, Guo ZH (1989). Pathogenicity test of Autoeciousness *Aspergillus parasilicus* on *Hemiberlesia pitysophila*.. Forest Science and Technology.

